# Transcriptome and *VcCADs* gene family analyses reveal mechanisms of blight resistance in rabbiteye blueberry

**DOI:** 10.3389/fpls.2025.1601658

**Published:** 2025-06-18

**Authors:** Shan-Shan Lu, Wei Liu, Li-Juan Yang, Cui-Lian Wu, Zhang-Yang Dai, Ming-Liang Zhu, Xuan Gao, Feng He, Jia-Xin Xiao, Bo Zhu

**Affiliations:** ^1^ Key Laboratory for the Conservation and Utilization of Important Biological Resources, College of Life Sciences, Anhui Normal University, Wuhu, Anhui, China; ^2^ Teaching and Research Group of Agronomy, Taihu Vocational and Technical School, Anqing, Anhui, China; ^3^ Planting Business Division of Ziyue Technology Group Co., Ltd., Wuhu, Anhui, China

**Keywords:** rabbiteye blueberry, *Neofusicoccum parvum*, lignin, cinnamyl alcohol dehydrogenase, shoot blight

## Abstract

**Introduction:**

Blueberry branch blight is a severe plant disease primarily caused by *Neofusicoccum parvum*.

**Methods:**

In this study, one-year-old branches of two rabbiteye blueberries (*Vaccinium virgatum*): resistant Tifblue ('TF') and susceptible Climax ('DF') were selected as experimental materials, the physiological parameters of two cultivars pre- and post- *N. parvum* inoculation. Transcriptomic analysis and comprehensive gene family characterization were conducted to examine the differential gene expression.

**Results:**

The results indicated that 'TF' exhibited higher lignin content and activity levels of peroxidase enzymes (POD), phenylalanine ammonia-lyase (PAL), and cinnamyl alcohol dehydrogenase (CAD) compared to 'DF'. RNA-sequence analysis revealed that at 24 hours post-inoculation, there were 136 and 121 differentially expressed genes related to lignin synthesis in 'TF' and 'DF', respectively. The analysis of these DEGs revealed that numerous of key enzyme genes in lignin synthesis pathways, including *PAL*, *4CL*, *CCR*, *CAD*, *HCT*, *COMT* and *POD* were annotated. Additionally, we characterized the *CAD* gene family, identifying 93 *CAD* coding genes at the blueberry genome level, which were classified into four subgroups. Most *CAD* gene promoters contained response elements associated with plant stress resistance pathways. QRT- PCR experiments demonstrated that the expression levels of *VcCAD8* and *VcCAD62* genes were significantly upregulated at 24 hours post-inoculation.

**Discussion:**

These findings suggest that *VcCADs* enhance lignin synthesis, improve the resistance of blueberries to shoot blight, and provide novel insights into the defense mechanisms of blueberries against *N. parvum*.

## Introduction

Blueberries (*Vaccinium* spp.), members of the Ericaceae family, are native to North America and are extensively cultivated for their high nutritional value, including anthocyanins, flavonoids, and other bioactive compounds (Strik and Yarborough, 2004). Recognized by the Food and Agriculture Organization (FAO) of the United Nations as one of the healthiest fruits, blueberries have gained global popularity for their health benefits (Wang et al., 2012b; Lorenzo et al., 2018; Edger et al., 2022). In 2024, China’s total blueberry planting area reached 95,880 hectares, with a production of 780,000 tons. The blueberry industry has significantly contributed to poverty alleviation and business development in China (Li et al., 2025). However, the expansion of blueberry cultivation has led to the emergence of various diseases, such as shoot blight (Kong et al., 2010), leaf spot (Tennakoon et al., 2018), and fruit rot (Polashock et al., 2009). Blueberry branch blight is a severe plant disease, which is mainly caused by fungi within the *Botryosphaericeae*. This pathogen invades plant vascular tissues through natural openings (such as lenticels, stomata) or wounds, leading to branch ulceration, shoot wilting, and eventual plant death (Wright and Harmon, 2010). The pathogen spreads through wind, rain, and insects, infecting a wide range of hosts, including blueberries, pomegranates, and grapes, causing symptoms such as leaf blight, shoot blight, and stem cankers (Restrepo-Leal et al., 2024). It is one of the most significant diseases in blueberry production, causing substantial economic losses (Wright and Harmon, 2010). Due to the rainy season in the middle and lower reaches of the Yangtze River, blueberry plants are more susceptible to mechanical damage, leading to wound infection. The spread of this pathogen could significantly impact on the blueberry industry (Xu et al., 2013). Studies have shown that *Neofusicoccum parvum* (*N. parvum*) can infect blueberry and cause symptoms of branch blight (Yu et al., 2012, 2013; Haenzi et al., 2021). Additionally, it is reported that many *N. parvum* isolates were isolated from horticultural plants, such as *Prunus pseudocerasus*, *Prunus persica*, *Latanus acerifolia*, *R.simsii* and *Vaccinium ashei*, which are widely cultivated in the field or along the road in China (He et al., 2022). Therefore, controlling the occurrence and spread of blueberry shoot blight is crucial in blueberry production. However, research on blueberry resistance to shoot blight remains limited, and studies on the disease resistance mechanisms of blueberries against shoot blight are urgently needed.

Lignin biosynthesis enhances plant defense by forming a physical barrier through cell wall lignification, which restricts the diffusion of pathogen-derived enzymes (such as cellulases) and toxins (Li et al., 2022a), and by providing chemical defense through antifungal lignin monomers and derivatives such as sinapyl alcohol (Zhang et al., 2024). The phenylpropanoid metabolic pathway governs lignin biosynthesis, with cinnamyl alcohol dehydrogenase (CAD) acting as the rate-limiting enzyme responsible for the reduction of hydroxycinnamaldehydes to monolignols (such as coniferyl alcohol) (Ng et al., 2024).

CAD is an important enzyme for lignin biosynthesis, which catalyzes the reduction of cinnamaldehydes into cinnamyl alcohol, the last step of monolignol biosynthesis before polymerization (Guo et al., 2010; Ma, 2010; Cesarino, 2022; Liao et al., 2025). Studies have demonstrated that overexpression of *DkCAD1* can significantly diminish the area of leaf lesions caused by persimmon anthracnose, concurrently with an increase in lignin content (Fan et al., 2024). Furthermore, research has shown that *CAD7* can suppress the pattern-triggered immunity (PTI) defense response in plants (Li et al., 2019b; Fan et al., 2024; Hua et al., 2025).

Although CAD pathways have been extensively characterized in model plants, specific research in blueberries is scarce (Rong et al., 2016; Park et al., 2018; Li et al., 2019a). In this study, we preliminarily identified the disease-resistant cultivar Tifblue (‘TF’) and the susceptible cultivar Climax (‘DF’) following infection with *N. parvum*. The relevant physiological indices of two cultivars with *N. parvum* infection were determined. The lignin content and key enzymes activity of lignin biosynthesis were significantly higher in ‘TF’ than ‘DF’, indicating the critical role of higher content of lignin in pathogen defense. Thus, we will employ RNA-sequencing (RNA-seq) and bioinformatics analysis to identify *VcCADs* gene family in blueberries and elucidate the association between enhanced lignification and pathogen control. This study aims to provide key molecular targets for resistance breeding and offer a theoretical framework for the development of blueberry cultivation and shoot blight resistance.

## Materials and methods

### Blueberry cultivation conditions and strain activation culture

**Figure 1:**
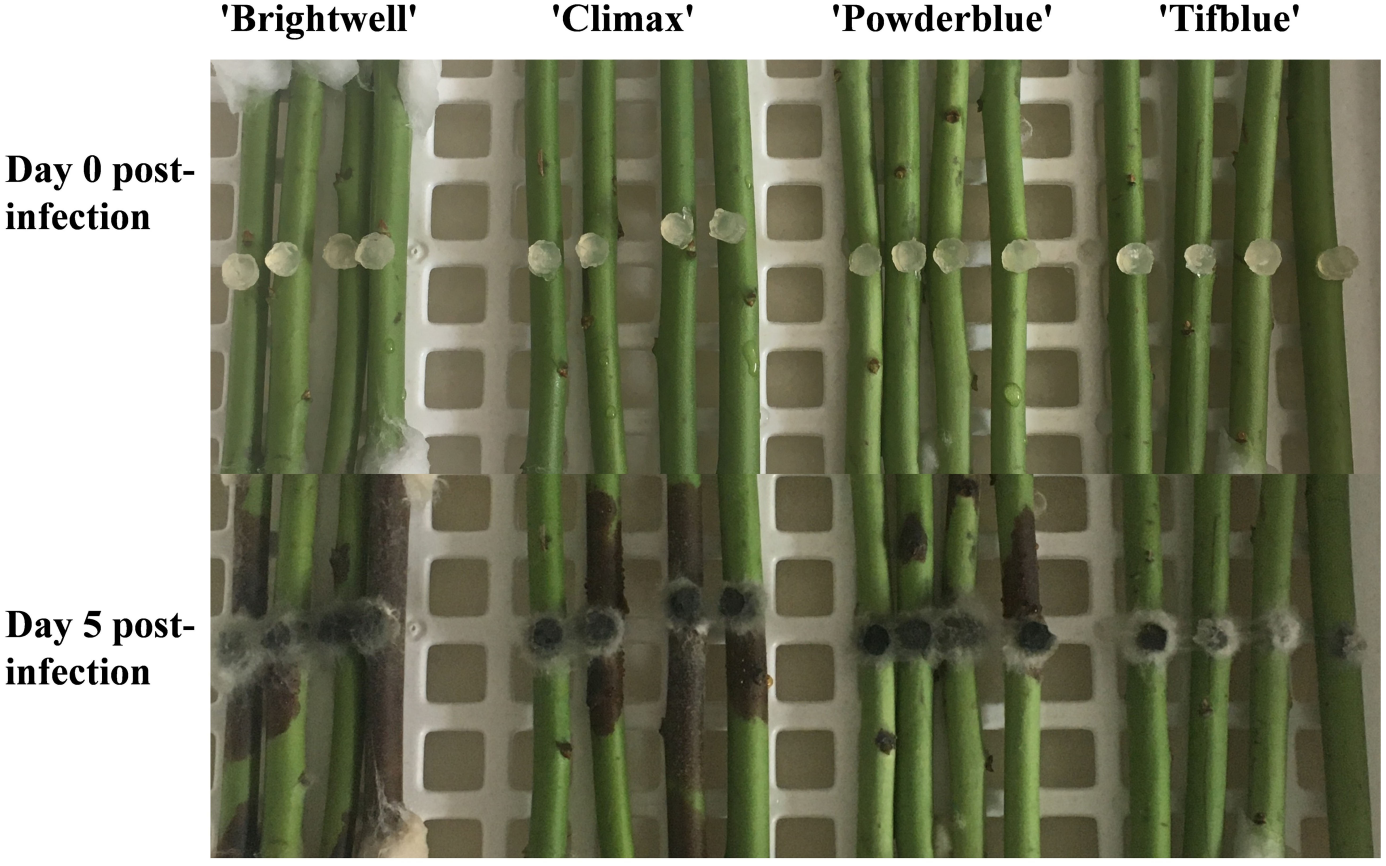
Symptoms of one-year-old branches of different blueberry varieties after *N. parvum* infection.

*N. parvum* was maintained in the Plant Physiology Section of Anhui Normal University (He et al., 2022). *N. parvum* was cultured in potato dextrose agar (PDA) plates supplemented with 50 mm ampicillin at 25°C for 2 days. A 5 mm diameter the agar plugs was inoculated on wounded one-year-old branches. One-year-old branches of four different rabbiteye blueberry varieties were collected on September 20 in 2021 at an organic of Anhui Huiking Agricultural Co., Ltd., Anhui province, China (118°08′E, 30°47N). Before inoculation, the branches were disinfected with 75% ethanol, rinsed with sterile water, and then air-dried on filter paper. The bark was wounded using a sterile iron rod (2 mm in diameter). A 5 mm agar block of *N. parvum* was placed on the wound. The ends of each branch were wrapped with wet cotton balls to maintain high relative humidity. All treated branches were placed in petri dishes lined with a layer of wet filter paper and covered with cling film. Four branches were inoculated per treatment, and the petri dishes were incubated at 25°C. We finally found that ‘TF’ was a resistant variety and ‘DF’ was a susceptible variety (**Figure 1**).

### Biochemical and lignin-related enzyme activity experiments in blueberries

One-year-old branches of two-year-old blueberry plants were selected to investigate the effects of *N. parvum* infection on the growth of blueberry plants. *N. parvum* was cultured in potato dextrose broth (PDB) and then harvested after two days. The branches of two-year-old blueberry plants were disinfested with 75% ethanol, washed with sterilized water, and dried on filter paper before inoculation. A 5 mm incision was made in the phloem of the disinfected part of the blueberry branch using a sterile scalpel. Cultured mycelia of uniform biomass were then evenly applied onto the wound sites. After 0, 1, 3, 5, and 7 days of infection, blueberry leaves and stems above the infected site of the branches were collected as experimental materials, and relevant physiological indices were measured.

Chlorophyll content was determined using ethanol extraction (Li, 2000). 0.1 g of blueberry young leaves were taken, 5 mL of 95% ethanol was added, and the supernatant was taken at 665 nm, 649 nm, and 470 nm to determine the absorbance after standing for 24 hours under dark conditions. The soluble protein content was determined using the Thomas Brilliant Blue B-250 method. The lignin content was extracted using the test kit (Beijing Solepol Technology Co., Ltd.; BC4200). The method for determining POD activity was based on Li (Li, 2000); Blueberry stem segments (0.2 g) from two-year-old plants were weighed and mixed with 1.6 mL of pre-cooled PBS phosphate buffer (50 mmol/L, pH 7.8). The mixture was homogenized using a grinding apparatus. The homogenate was transferred to a centrifuge tube. The tube was centrifuged at 4°C and 12,000 r for 20 minutes. After centrifugation, the supernatant was carefully collected for further use. To determine the activities of PAL and CAD, follow the instructions of PAL and CAD assay Kits (Beijing Regen Biotechnology Co., Ltd., TE040350T and TE041925T) respectively. This experiment was performed three times with three biological replicates of each treatment.

### Screening and analysis of differentially expressed genes transcriptome in blueberry

For transcriptome sequencing, *N. parvum* was cultured as described for the physiological indices. One-year-old branches of ‘TF’ and ‘DF’ blueberry were disinfested with 75% ethanol, washed with sterilized water, and dried on filter paper before inoculation. A 5 mm incision was made in the phloem of the one-year-old blueberry branches using a sterile scalpel. Cultured mycelia of uniform biomass were then evenly applied onto the wound sites. Tissue and mycelia were collected at 0 and 24^th^ hours post-infection and stored at -80°C for subsequent experiments. Four branches were inoculated per treatment, and the petri dishes were incubated at 25°C. Three biological replicates were collected for both varieties. Transcriptome sequencing was performed by Shenzhen Riokangchen Biotechnology Company, generating a total of 258,482,477 raw reads. After quality control, 253,197,179 clean reads were retained for downstream analysis. The blueberry whole genome sequence (Vdarrowii_genome.v2.4-gene.fasta) was downloaded from the GDV website (https://www.vaccinium.-org/data_download) as the reference genome, and the FPKM value of each gene was used as the expression quantification to screen for differential genes using P-value < 0.001 of FDR < 0.05.

### Identification of *CAD* gene families in blueberry

Nine *Arabidopsis thaliana* CAD protein sequences were obtained from Ensembl Plants (https://plants.ensembl.org/index.html) and served as queries for BLASTP searches against our local transcripts databases, with a threshold of 1×10–^5^ to filter the results. Furthermore, the CAD domains (PF08240 and PF00107) retrieved from the Pfam database (http://pfam-legacy.xfam.org/) were employed to generate an HMM file using HMMER3.4. Local protein databases were queried with this HMM using HMMER3.4, applying a threshold of 0.01 to ensure the inclusion of sequences highly probable to belong to the CAD domain family. Each candidate CAD-related protein sequence was validated as a member of the *CAD* gene family using InterPro (https://www.ebi.ac.uk/interpro/) and the Conserved Domain Database (CDD) (https://www.ncbi.nlm.nih.gov/Structure/bwrpsb/bwrpsb.cgi). Candidate genes lacking CAD domains or with incomplete domain structures were ultimately excluded from the dataset.

### Construction of phylogenetic trees

Blueberry CAD proteins were aligned using MAFFT (available at http://mafft.cbrc.jp/alignment/server/, accessed on 25 September 2019). A CAD phylogenetic tree was constructed using IQ-TREE version 1.6.8 (Nguyen et al., 2015). A CAD phylogenetic tree was constructed with the Maximum Likelihood (ML) method. Bootstrap analysis was performed with 1000 replicates to assess the tree’s robustness. The resultant trees were visualized and, where necessary, manually refined using the online tool iTOL.v7 (https://itol.embl.de/).

### Collinearity analysis, chromosome localization and Ka/Ks of gene duplication

Following transcriptome sequencing, the GFF3 (General Feature Format version 3) genome annotation files and *CAD* gene family member ID list were analyzed using the MapGene2Chrom web tool (http://mg2c.iask.in/mg2c_v2.0/) to generate chromosomal location maps, and visualize gene positional information on chromosomes using TBtools. Intraspecies collinearity analysis was performed using the One Step MCScanX module in TBtools software, with the blueberry genome sequence files and gene annotation files as input. A circos plot was subsequently generated to visualize the collinear relationships within the blueberry species. Ka/Ks values, derived from the Simple Ka/Ks Calculator in TBtools software, were used to analyze the evolutionary pressure on the *CAD* gene family.

### QRT-PCR validation of *CAD* family member genes

To study the expression analysis of blueberry *CAD* family member genes, we took samples and extracted RNA of ‘TF’ and ‘DF’ one-year-old branches inoculated with *N. parvum* after 0 and 24^th^ hours. RNA was extracted using the RNA extraction kit (TaKaRa), and the cDNAs were synthesized using the qPCR RT Master Mix (TaKaRa). Blueberry *CAD* mRNA sequences were searched in our transcript database, and quantitative primers were designed using Primer 3.0 (**Table 1**). Quantitative results were calculated based on the CT (Threshold cycle) values of a blueberry metallothionein gene (*NA186*) (Walworth et al., 2012) as the internal reference gene to be detected using the 2^-ΔΔCT^ method. The experiment was performed three biological repetitions for each sample.

**Table 1:** PCR primer used for studying of genes under test.

Gene name	Forward primer	Reverse primer	Tm value/°C
*NA186*	ACCCTGACATGAGCTTCTCG	ACCCAAATCTCTGCTTGCTG	59.7
*VcCAD8*	GTATGTGAATGAAGCTATTGAGAGG	CACTTCAAGGAGTTCTCGATGT	57.8
*VcCAD19*	CTGCAGTTCACTCTCTCATGC	GTCCAATGTTATGTTGTGCTTCG	58.8
*VcCAD21*	CGATGGATTATGTGAACACTGC	GTTTGCAACATCAAGGACGAA	57.7
*VcCAD58*	CCATTTTCCCTTTGCTTGTTGG	TCTATCATTTCTTGCGTCTCCTTC	58.7
*VcCAD62*	AATTTCAGGGAGTGCAGCAGG	CAACCTCTCAATAGCTTCATTCAC	57.9
*VcCAD68*	TGGACTATGTGAACACTGCAATG	AGTCGACATGATCTCCTTTCCA	58.9
*VcCAD72*	ACTTCTGTGCTGCAAACAAGA	CACTTCAAGGAGTTCTCGATGT	58.1
*VcCAD93*	AATTTCAGGGAGTGCATCAGG	CTGGGTAAATCTTGTTTGCAGC	58.3

### Data analysis

The data obtained from the experiments were analyzed by one-way ANOVA using SPSS 26.0 (Statistical product and service solutions, USA) software; plotted using Excel 2019 and GraphPad Prism 8.0; and analyzed by Draw Veen Diagram Ranges of the Veen Diagram by differential gene analysis and heatmap by TBtools.

## Results

### Differences in physiological parameters and lignin synthesis-related enzyme activities between two varieties after inoculation with *N. parvum*

**Figure 2:**
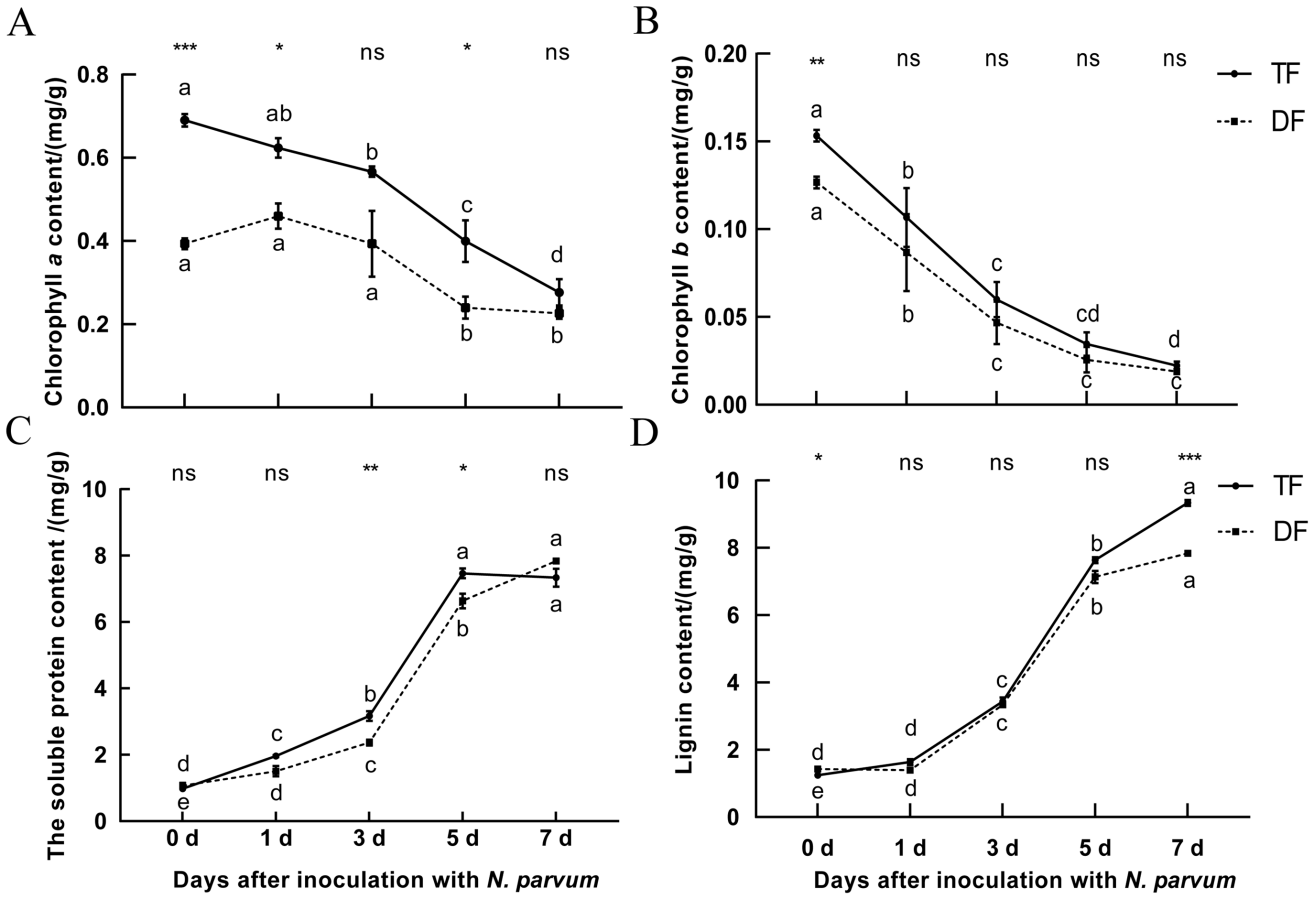
Effect of inoculation with *N. parvum* on chlorophyll content, soluble protein content, and lignin content in blueberries. **(A)** chlorophyll a content, **(B)** chlorophyll b content, **(C)** soluble protein content, **(D)** lignin content. Values are means ± SD (n = 4). Differences in comparisons between data from the same variety of blueberry inoculated at different times were tested using Duncan's method of one-way ANOVA (one-way ANOVA) with a significance level of P < 0.05. The same lowercase letters indicate no significant difference as determined by Duncan's test (P ≥ 0.05), while different lowercase letters indicate significant differences (P < 0.05).Data from different varieties of blueberries with the same inoculation time were processed using t-test, *P < 0.05, **P < 0.01, ***P < 0.001, Student's t-test. ns indicates no significant difference. 'TF', 'Tifblue' blueberry; 'DF', 'Climax' blueberry. Two-years-old plants were used as sample. The experiment was repeated three biological replicates.

Chlorophyll content is a critical indicator to evaluate the efficiency of plant photosynthesis and growth status (Herritt et al., 2020). In this study, the chlorophyll content of ‘TF’ and ‘DF’ blueberries inoculated with *N. parvum* was determined in a time gradient. Both chlorophyll a and chlorophyll b contents decreased in both varieties, with significant reductions observed on the 3^rd^ and 7^th^ days post-inoculation for chlorophyll a, and on the 5^th^ day for chlorophyll b (**Figures 2A, B**). This indicates that inoculation with *N. parvum* can impact the chlorophyll content of blueberry leaves and that blueberry susceptible varieties showing lower chlorophyll content than resistant varieties.

Soluble protein content is an important physiological marker of overall plant metabolism (Wang et al., 2012a). Our results showed that the soluble protein content of blueberry increased over time after inoculation with *N. parvum.* The peak soluble protein content in ‘TF’ blueberries occurred on the 5^th^ day, whereas in ‘DF’ blueberries, it peaked on the 7^th^ day, with ‘TF’ blueberries exhibiting lower levels than ‘DF’ blueberries (**Figure 2C**). This suggests that more enzymes are involved in the defense response of disease-resistant blueberry varieties during the process. Interestingly, we observed a transient increase in chlorophyll a content in the susceptible ‘DF’ variety on the first day of infection, while the resistant ‘TF’ variety showed a decline in soluble protein content when it reached its peak.

Lignin is a key component of plant secondary cell walls and serving important functions such as water transportation, mechanical support, and resistance to pathogens (Rogers et al., 2005). Blueberry lignin content increased over time after inoculation with *N. parvum*. By the 7^th^ day, ‘TF’ blueberries had significantly higher lignin content than ‘DF’ blueberries (**Figure 2D**). This suggests that blueberries may defend against pathogenic microorganisms by increasing lignin content when inoculation with *N. parvum* and that the ‘TF’ blueberries lignin content of disease-resistant blueberry was higher than that of disease-susceptible ‘DF’ blueberries. These results indicated that the growth and development of blueberry were affected after inoculation with *N. parvum*, and the resistance of disease-resistant varieties to the pathogen maybe higher than that of susceptible blueberry.

**Figure 3:**
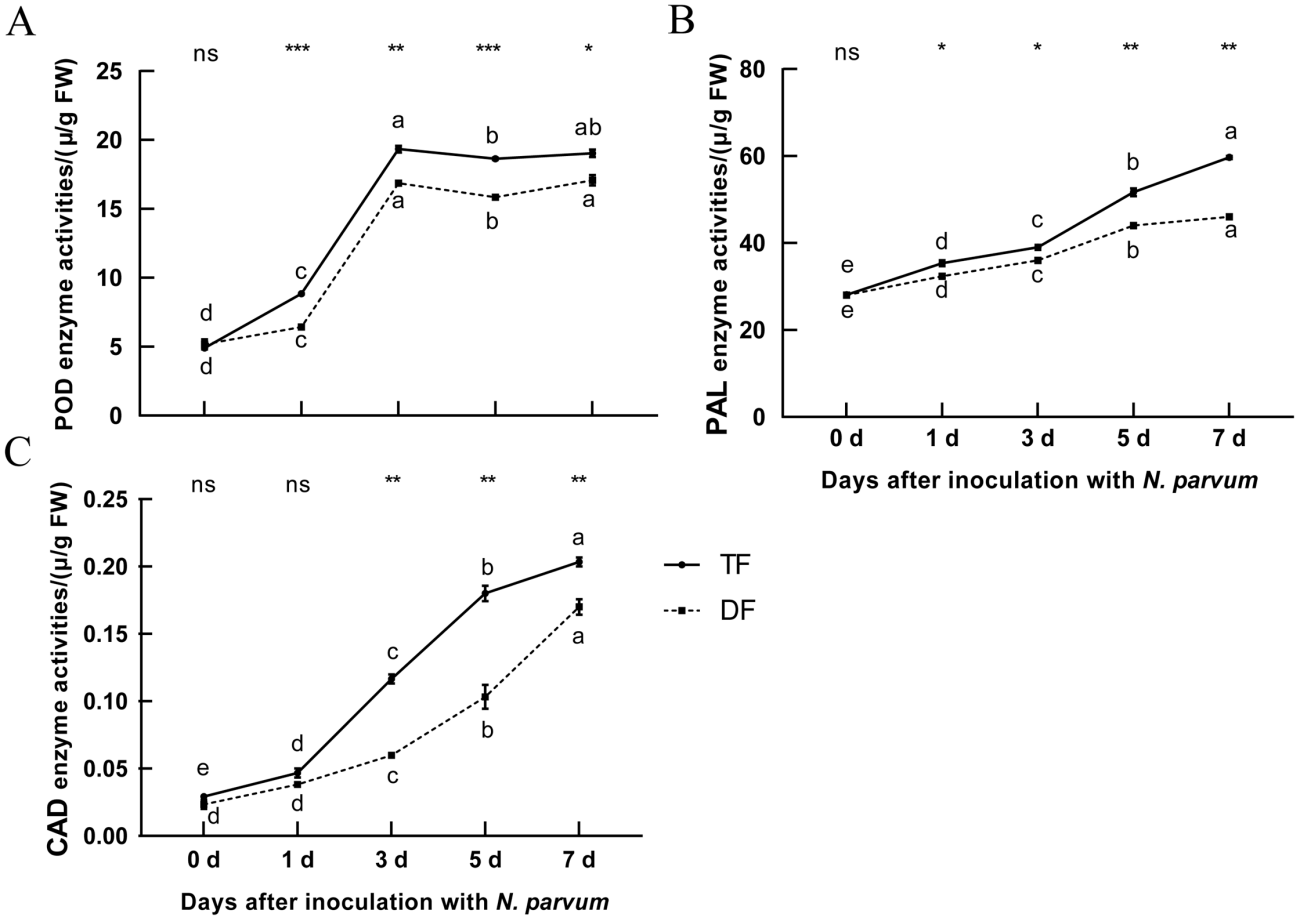
Effect of inoculation with *N. parvum* on POD, PAL, and CAD enzyme activities of blueberry. **(A)** POD, **(B)** PAL, **(C)** CAD. The note was the same to **Figure 2**. Differences in comparisons between data from the same variety of blueberry inoculated at different times were tested using Duncan's method of one-way ANOVA (one-way ANOVA) with a significance level of P < 0.05. The same lowercase letters indicate no significant difference as determined by Duncan's test (P ≥ 0.05), while different lowercase letters indicate significant differences (P < 0.05). Data from different varieties of blueberries with the same inoculation time were processed using t-test, *P < 0.05, **P < 0.01, ***P < 0.001, Student's t-test. ns indicates no significant difference. 'TF', 'Tifblue' blueberry; 'DF', 'Climax' blueberry. Two-years-old plants were used as sample. The experiment was repeated three biological replicates.

Blueberry lignin derivatives enhance both the physical and chemical defense mechanisms of the plant by activating the expression of defense-related genes (Qu et al., 2022). CAD, PAL, and POD are key enzymes in the lignin synthesis pathway. The results showed that the contents of CAD, PAL, and POD in blueberries increased significantly following *N. parvum* inoculation (**Figure 3**), and the lignin synthesis-related enzyme activities observed in disease-resistant varieties compared to disease-susceptible ones (**Figure 3**). This suggests an increase in the activity of related enzymes in lignin synthesis in blueberries to inoculation with *N. parvum*.

### Involvement of the phenylpropane biosynthetic pathway in the resistance of blueberries after inoculation with *N. parvum*

**Figure 4:**
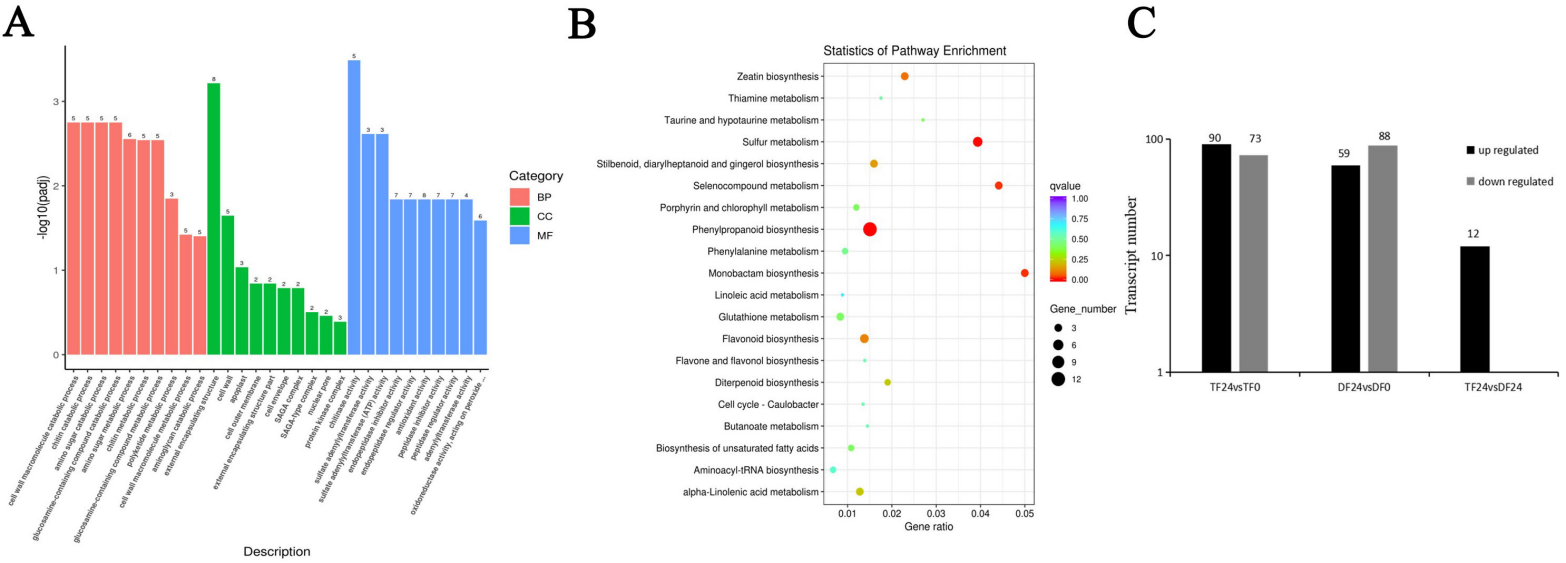
Analysis of DEGs of blueberry inoculated with *N. parvum*. **(A)** gene ontology (GO) functional classification of top 30 differently expressed genes in the comparison of in 'TF'24 vs 'DF'24, **(B)** the first 20 ways of KEGG enrichment analysis of differential genes in the comparison of in 'TF'24 vs 'DF'24, **(C)** number of differentially expressed genes in phenylpropanoid biosynthesis pathway ('TF'24 vs 'TF'0, 'DF'24 vs 'DF'0 and 'TF'24 vs 'DF'24).

Next, we analyzed the transcriptome data and found a large number of differentially expressed genes (DEGs) up-regulated or down-regulated in both ‘TF’ and ‘DF’ blueberries after inoculation with *N. parvum* for 24 hours. We further analyzed these DEGs using Gene Ontology (GO) functional enrichment. The results revealed that the biological processes primarily involved cell wall macromolecule catabolic processes, chitin catabolic processes, and amino sugar metabolic processes, with amino sugar metabolism being the most frequently observed among the differentially expressed genes. The cellular components mainly included the external encapsulating structure, cell wall, and apoplast, with the largest number of genes associated with the extracellular encapsulating structure. The main biological functions included chitinase activity, sulfate adenylyltransferase activity, sulfate adenylyltransferase (ATP) activity, and antioxidant activity, with antioxidant activity being the most differentially expressed gene (**Figure 4A**). This suggested that after the blueberry was inoculated with *N. parvum*, the cell wall macromolecule decomposition metabolic process was the most obvious in the biological process, the external encapsulating structure was the most different in the cellular components, and the activity of chitinase was the most different in the biological function. The most DEGs were found in the extracellular encapsulating structures, indicating that many biochemical reactions occur in blueberry cells after infection, particularly affecting cell wall components. After blueberries inoculation with *N. parvum*, a series of enzymes were involved in the reaction, including peroxidase, which is also a key enzyme in the lignin synthesis pathway.

The major differential pathways in Kyoto Encyclopedia of Genes and Genomes (KEGG) enrichment analysis included phenylpropanoid biosynthesis, sulfur metabolism, monobactam biosynthesis, selenocompound metabolism, zeatin biosynthesis. The results showed that the phenylpropanoid biosynthesis pathway exhibited the most significant differences, involving the highest number of genes, and was the most critical for blueberry resistance to *N. parvum* infection (**Figure 4B**). We further analyzed the differential genes between ‘TF’ and ‘DF’ after inoculating with *N. parvum* at 0 and 24^th^ hours post-inoculation. The results showed that there were 90 up-regulated genes and 73 down-regulated genes in ‘TF’ 24 vs ‘TF’ 0; in ‘DF’ 24 vs ‘DF’ 0, there were 59 up-regulated genes and 88 down-regulated genes. While in ‘TF’ 24 vs ‘DF’ 24, there were only 12 up-regulated genes (**Figure 4C**). These findings suggested that after inoculation with *N. parvum*, the number of DEGs in both ‘TF’ and ‘DF’ varieties increased.

### Involvement of blueberry lignin synthesis in response to inoculation with *N. parvum*

Four lignin metabolic pathways can be monitored from the phenylpropane metabolic pathway, including p-Hydroxy-phenyl lignin, Guaiacyl lignin, 5-Hydroxy-guaiacyl lignin, and Syringyl lignin (Zhong et al., 2010). 16 genes were included in the phenylpropanoid biosynthesis pathway. *Cyp98A* and *C3’H* genes were only upregulated or downregulated in ‘TF’ 24 vs ‘TF’ 0, while *CCoAOMT*, *CYP84A*, and *F5H* genes were only downregulated in ‘DF’ 24 vs ‘DF’ 0 (**Table 2**).

**Table 2:** Differentially expressed genes in phenylpropanoid biosynthesis pathway of blueberry inoculated with *N. parvum*.

KO	Symbol	Name	UniGenes		
			'TF'24 vs 'TF'0	'DF'24 vs 'DF'0	'TF'24 vs 'DF'24
K10775	PAL	phenylalanine ammonia-lyase	↓	↓	↑
K00487	CYP73A	trans-cinnamate 4-monooxygenase	↑	↑	/
K13066	COMT	caffeic acid 3-O-methyltransferase / acetylserotonin O-methyltransferase	↓	↓	/
K01904	4CL	4-coumarate--CoA ligase	↓	↓	/
K13065	HCT	shikimate O-hydroxycinnamoyltransferase	↑、↓	↑、↓	↑
K09754	CYP98A, C3'H	5-O-(4-coumaroyl)-D-quinate 3'-monooxygenase	↑、↓	/	/
K09753	CCR	cinnamoyl-CoA reductase	↑	↑	
K12355	REF1	coniferyl-aldehyde dehydrogenase	↑、↓	↑、↓	↑
K00083	CAD	cinnamyl-alcohol dehydrogenase	↑、↓	↑、↓	↑
K00430	POD	peroxidase	↑、↓	↑、↓	↓
K11188	PRDX6	peroxiredoxin 6	/	/	/
K09755	CYP84A, F5H	ferulate-5-hydroxylase	/	↓	/
K00588	CCoAOMT	caffeoyl-CoA O-methyltransferase	/	↓	/
K01188	E3.2.1.21	beta-glucosidase	↓	↑、↓	/
K05349	bglX	beta-glucosidase	↑、↓	↑、↓	/
K05350	bglB	beta-glucosidase	↑、↓	↑、↓	/

The symbol ↓ indicates gene downregulation, and the symbol ↑ indicates gene upregulation.

**Figure 5:**
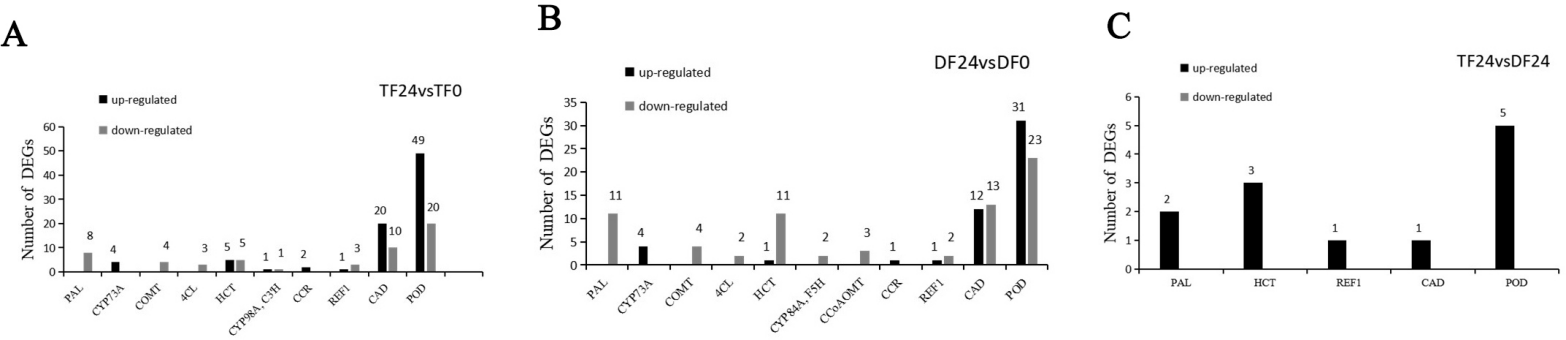
Differentially expressed genes related to lignin biosynthetic pathway in blueberry inoculated with *N. parvum*. **(A)** 'TF'24 vs 'TF'0, **(B)** 'DF'24 vs 'DF'0, **(C)** 'TF'24 vs 'DF'24.

To elucidate the phenylpropanoid biosynthesis pathway, we conducted a comparative analysis of the transcriptomes of distinct blueberry varieties inoculated with *N. parvum* to identify differential genes related to lignin synthesis. The results showed a total of 136 DEGs in ‘TF’ 24 vs ‘TF’ 0. Among these, 69 genes were related to POD enzyme activity, including 49 up-regulated and 20 down-regulated genes. There were 30 *CAD* enzyme synthesis-related genes, of which 20 were up-regulated and 10 down-regulated genes. Additionally, 10 genes were related to hydroxycinnamoyl-CoA:shikimate/quinate hydroxycinnamoyltransferase (HCT) enzyme activity, with five genes up-regulated and five down-regulated. There were four *REF1* enzyme-related genes, including one up-regulated and three down-regulated expression genes, and two cytochrome P45098A (*CYP98A*) enzyme related genes, including one up-regulated and one down-regulated gene (**Figure 5A**). The results showed a total of 121 DEGs in ‘DF’ 24 vs ‘DF’ 0. There were 54 *POD* enzyme-related genes, including 31 up-regulated and 23 down-regulated genes. There were 25 *CAD* enzyme synthesis-related genes, including 12 up-regulated and 13 down-regulated genes. There were 12 *HCT* enzyme-related genes, one up-regulated and 11 down-regulated genes, and three *REF1* enzyme related genes, one up-regulated and two down-regulated genes (**Figure 5B**). In the comparison of ‘TF’ 24 vs ‘DF’ 24, there were a total of 12 DEGs, all of which were up-regulated (**Figure 5C**). The above results indicated that a large number of lignin synthesis pathway genes were up-regulated in ‘TF’-resistant and ‘DF’-susceptible varieties after infection by *N. parvum*, mainly *CAD* and *POD*-related genes.

**Figure 6:**
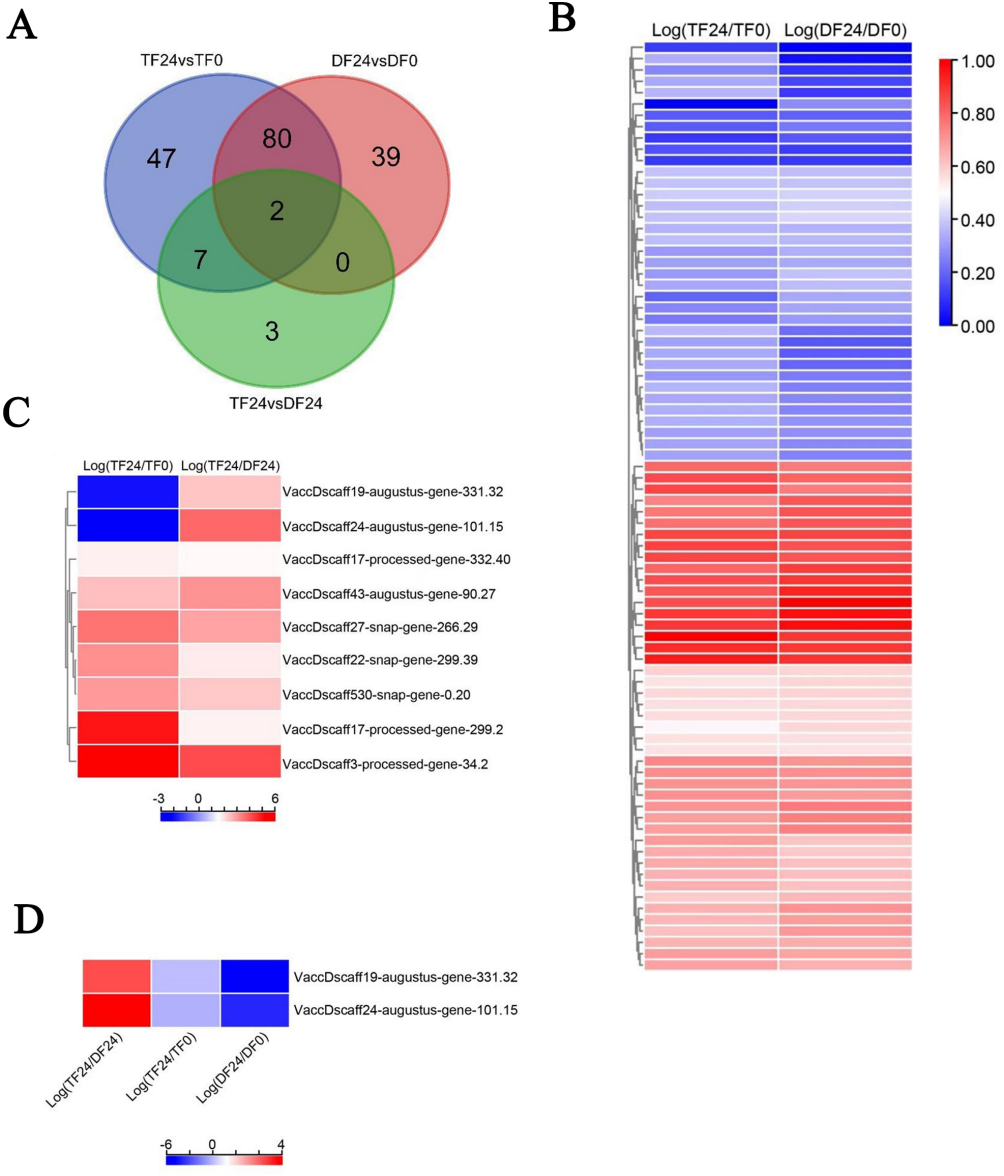
Analysis of DEGs of lignin synthesis pathway in blueberry. **(A)** the Wayne diagram, **(B)** the heat map of DEGs between 'TF'24 vs 'TF'0 and 'DF'24 vs 'DF'0, **(C)** the heat map of DEGs between 'TF'24 vs 'TF'0 and 'TF'24 vs'DF'24, **(D)** the heat map of DEGs among 'TF'24 vs 'TF'0, 'DF'24 vs 'DF'0 and 'TF'24 vs 'TF'24.

We next analyzed expression differential genes related to the lignin synthesis pathway in blueberry. The Venn diagram results showed that 82 common DEGs were found in the two comparative combinations of 'TF' 24 vs 'TF' 0 and 'DF' 24 vs 'DF' 0 (**Figure 6A**). Among 82 DEGs, 37 genes were down-regulated, and were mainly annotated in the transcriptome as encoding enzymes related to lignin synthesis, such as CAD, PAL, and aldehyde dehydrogenase (**Figure 6B**). In the two comparative combinations of 'TF' 24 vs 'TF' 0, and 'TF' 24 vs 'DF' 24, there were nine common DEGs, among which seven genes were up-regulated and mainly annotated in the transcriptome as encoding peroxidases, dehydrogenases, and transferases. In addition, two genes were downregulated and annotated in the transcriptome as encoding PAL enzymes. No consistently expressed genes were found in the comparison combinations of 'DF' 24 vs 'DF' 0, and 'TF' 24 vs 'DF' 24 (**Figure 6C**). In the three comparative combinations of 'TF' 24 vs 'TF' 0, 'DF' 24 vs 'DF' 0, and 'TF' 24 vs 'DF' 24, two DEGs were annotated as encoding PAL enzymes in the transcriptome (**Figure 6D**).

In summary, the DEGs revealed that key enzyme genes in lignin synthesis pathways, such as *PAL*, *4CL*, *CCR*, *CAD*, *HCT*, *CoMT*, and *POD*.

### Bioinformatics analysis of blueberry *CAD* family genes

**Figure 7:**
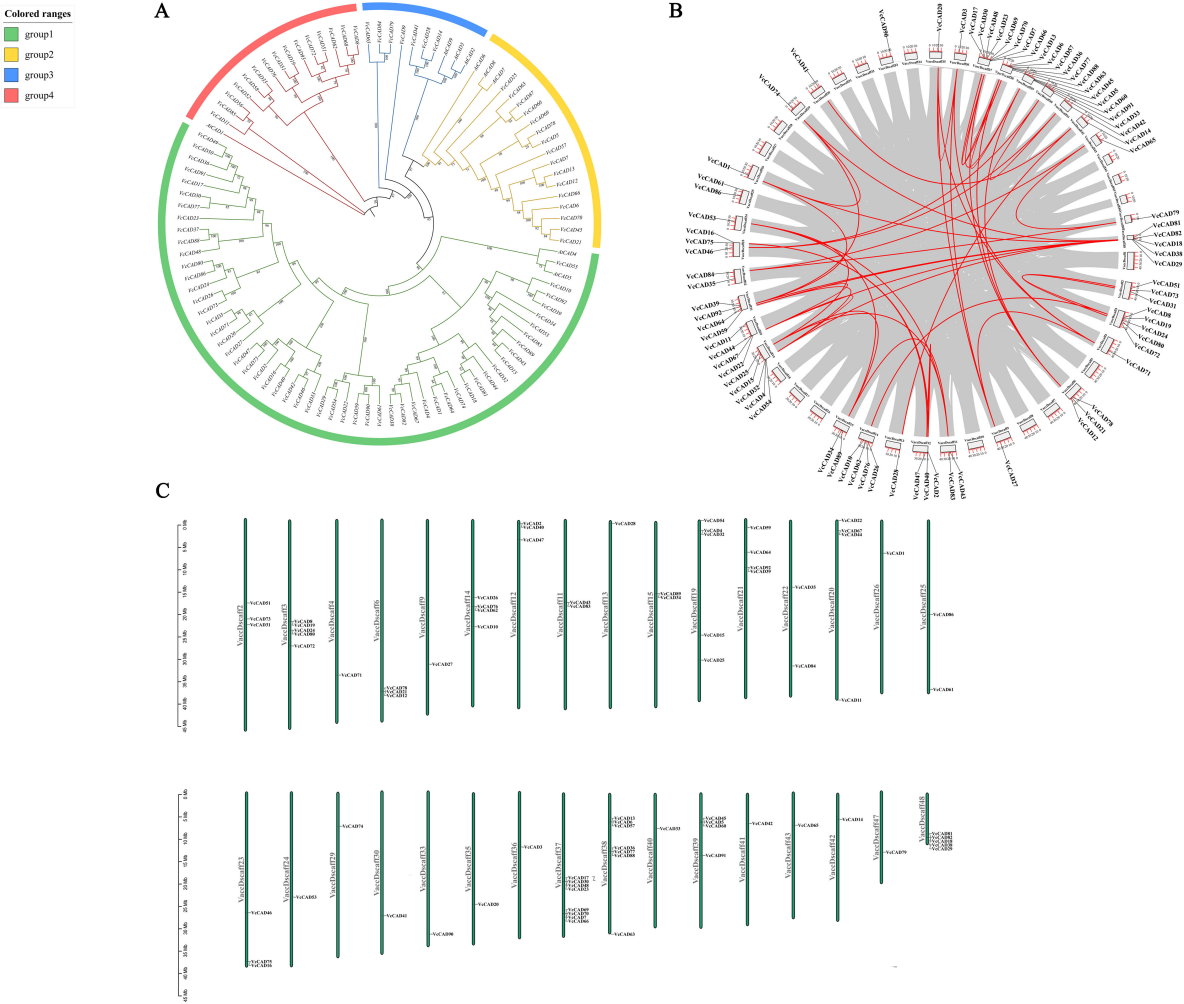
Identification of blueberry *CAD* gene family and physicochemical characterization of encoded proteins. **(A)** the phylogenetic tree, **(B)** collinear analysis, **(C)** chromosome mapping of members.

*CAD* genes are involved in the final step of the lignin biosynthesis reaction and play an important role in plant resistance to biotic stresses such as pathogens through the lignin pathway (Sibout et al., 2005). Our study found that the lignin content and CAD enzyme activity of blueberries were significantly elevated after inoculation with *N. parvum*, suggesting that the *CAD* gene may play an important role in the resistance of blueberries to *N. parvum* infection. Nine reported *CAD* genes of *Arabidopsis thaliana* were phylogenetically analyzed in combination with *CAD* genes of blueberry, which divides the phylogenetic tree of *CAD* genes of *Arabidopsis thaliana* and blueberry into a total of four groups 1, 2, 3, and 4. Group 1 contained two *AtCADs* (*AtCAD6*, *AtCAD5*) and 55 *VcCADs*; Group 2 contained three *AtCAD* (*AtCAD6*, *AtCAD7*, *AtCAD8)* and 16 *VcCADs*; Group 3 contained three *AtCAD* (*AtCAD2*, *AtCAD3*, *AtCAD9*) and seven *VcCADs*; and Group 4 had only one *AtCAD* (*AtCAD1*) and 15 *VcCADs* (**Figure 7A**). We analyzed the intragenomic collinearity relationships of the blueberry *CAD* gene family based on the blueberry genome annotation file and the genome sequence file. The results showed that, among the 93 *CAD* genes identified by the gene family, except for *VcCAD10*, *VcCAD11*, *VcCAD13*, *VcCAD19*, *VcCAD23*, *VcCAD24*, *VcCAD26*, *VcCAD30*, *VcCAD44*, *VcCAD48*, *VcCAD57*, *VcCAD60*, *VcCAD61*, *VcCAD62*, *VcCAD66*, *VcCAD67*, *VcCAD73*, *VcCAD77*, *VcCAD80*, *VcCAD86*, *VcCAD88*, and *VcCAD90*, the remaining genes were involved in segmental duplications. Moreover, the remaining 70 *CAD* genes formed 46 gene collinear pairs, and a tandem duplication event occurred between *VcCAD2* and *VcCAD47* on chromosome VaccDscaff12 (**Figure 7B**). The collinearity analysis indicated that the duplication pattern of *VcCAD* genes was mainly whole-genome duplication (WGD)/segmental duplication, suggesting that gene segment duplication was the main cause of the expansion of the blueberry *CAD* gene family during evolution. In addition, we also analyzed the distribution of blueberry *CAD* family genes on chromosomes. The results showed that 93 *CAD* genes were distributed on 32 chromosomes, and most of the *CAD* genes were located on chromosomes VaccDscaff37 and VaccDscaff38. Chromosomes VaccDscaff2, VaccDscaff3, VaccDscaff6, VaccDscaff12, VaccDscaff14, VaccDscaff19, VaccDscaff20, VaccDscaff21, VaccDscaff23, VaccDscaff37, VaccDscaff38, VaccDscaff39, and VaccDscaff48 all contained more than two genes arranged in gene clusters. Each of the 15 *CAD* genes, including *VcCAD1*, *VcCAD3*, *VcCAD14*, *VcCAD20*, *VcCAD27*, *VcCAD28*, *VcCAD33*, *VcCAD41*, *VcCAD42*, *VcCAD53*, *VcCAD65*, *VcCAD71*, *VcCAD74*, *VcCAD79*, and *VcCAD90*, was distributed as a single gene on the chromosomes (**Figure 7C**). To assess the evolutionary relationships and selection pressures within the *CAD* gene family, we calculated the Ka, Ks values, and Ka/Ks ratios for 50 homologous gene pairs (**Supplementary Table S1**). Among the analyzed pairs, 98% exhibited Ka/Ks ratios less than 1 (range: 0.062–0.629), indicating that the *CAD* family has primarily undergone purifying selection. The *VcCAD70*-*VcCAD6* pair showed the lowest Ka/Ks value (0.062), suggesting strong functional conservation. The only exception was the *VcCAD51*-*VcCAD72* pair (Ka/Ks = 1.973), where the nonsynonymous substitution rate was significantly higher than the synonymous rate (Ka = 0.022 vs Ks = 0.011), implying potential positive selection acting on this pair.

### Expression analysis of the *VcCADs* gene in response to resistance of blueberry after inoculate with *N. parvum*

**Figure 8:**
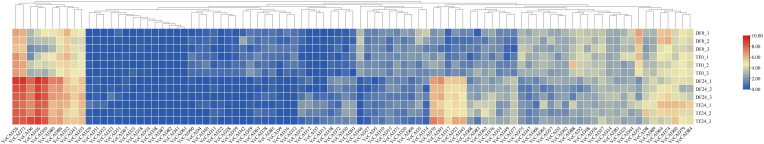
The heatmap showing expression analysis of *VcCADs* family genes of blueberry inoculated with *N. parvum*.

**Figure 9:**
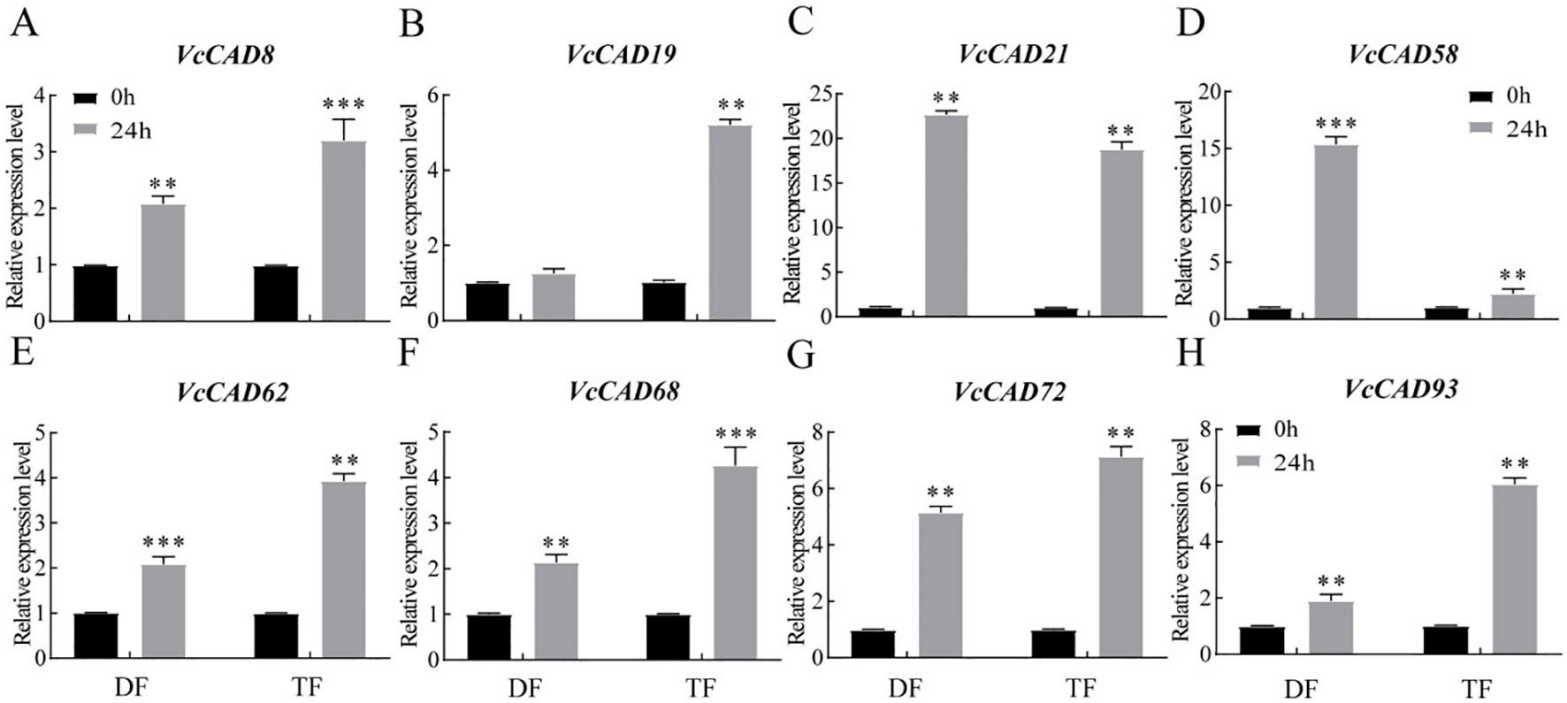
Expression analysis of the *VcCAD* genes were determined by qRT-PCR of blueberry inoculated with *N. parvum*. **(A)** *VcCAD8*, **(B)** *VcCAD19*, **(C)** *VcCAD21*, **(D)** *VcCAD58*, **(E)** *VcCAD62*, **(F)** *VcCAD68*, **(G)** *VcCAD72*, **(H)** *VcCAD93*. Data from different varieties of blueberries with the same inoculation time were processed using t-test, **P < 0.01, ***P < 0.001.

The results showed that the expression *VcCAD8*, *VcCAD19*, *VcCAD21*, *VcCAD58*, *VcCAD62*, *VcCAD68*, *VcCAD72* and *VcCAD93* were different in both in ‘TF’ and ‘DF’ blueberries after inoculated with *N. parvum* (**Figure 8**). We next verified the expression of 8 *VcCADs* genes by QRT-PCR. It was found that *VcCAD8*, *VcCAD19*, *VcCAD58*, *VcCAD62*, *VcCAD68* and *VcCAD93* expression were all elevated in both ‘TF’ and ‘DF’ blueberries following *N. parvum* inoculation (**Figure 9**).

## Discussion

### Inhibition of blueberry growth and development following inoculation with *N. parvum*

Chlorophyll, as the pivotal molecule for light energy capture in photosynthesis (Khangura et al., 2020), exhibits content variations that are directly correlated with plant growth and metabolic processes (Yuan et al., 2018). This study demonstrates that Vaccinium plants inoculated with Pestalotiopsis microspora exhibited significant chlorophyll depletion, potentially attributable to pathogen-induced damage in the photosynthetic apparatus. Notably, the susceptible cultivar ‘DF’ exhibited more pronounced chlorophyll reduction compared to the resistant cultivar ‘TF’ (**Figures 2A, B**). However, the transient increase in chlorophyll a content in susceptible cultivar ‘DF’ may be because during the initial stage of pathogen infection, plants initiate defense responses (such as synthesizing disease-resistant related substances and activating signaling pathways), which require a substantial amount of energy. As a core pigment in the light reaction, the transient increase in chlorophyll a may be a strategy for plants to enhance light energy capture efficiency and rapidly generate ATP and NADPH to meet the energy demands of defense metabolism. Such chlorophyll diminution likely impairs photosynthetic efficiency, thereby adversely affecting plant development (Wang et al., 2022).

Concomitantly, a marked elevation in soluble protein content was observed post-inoculation, indicative of plant stress responses against pathogen invasion. Intriguingly, the susceptible ‘DF’ cultivar displayed higher soluble protein accumulation than its resistant counterpart ‘TF’ (**Figure 2C**), possibly reflecting intensified metabolic activities during disease resistance processes in ‘DF’. A transient increase in chlorophyll a content was detected in ‘DF’ during the initial infection phase (day 1 post-inoculation), while ‘TF’ manifested a paradoxical decrease in soluble protein content at its peak accumulation phase. These phenomena suggested distinct defense strategies: the susceptible ‘DF’ might transiently enhance chlorophyll biosynthesis as an early stress response, yet subsequently suffer chloroplast degradation due to overloaded defense systems. In contrast, the resistant ‘TF’ likely employs a resource allocation strategy by downregulating non-essential protein synthesis to prioritize defense-related metabolic pathways.

These findings collectively suggested that *N. parvum* infection exerts multifaceted impacts on blueberry physiology, extending beyond chlorophyll reduction to metabolic reprogramming. The synergistic effects of chlorophyll depletion and soluble protein overaccumulation may collectively compromise normal physiological functions in host plants (Ye et al., 2025).

### Blueberry lignin synthesis pathway is involved in resistance to inoculation with *N. parvum*

Lignin is involved in the construction of plant disease defense systems (Wang et al., 2025). When pathogens infect plants, the content of lignin will increase, and the enzymes related to lignin synthesis will also change accordingly. When melons were inoculated with *Fusarium oxysporum*, their lignin content was significantly elevated and CAD enzyme activity was significantly increased in roots, stems and leaves (Liu et al., 2016). PAL is the first key enzyme in the lignin synthesis pathway. When plants are infected by pathogens, the activity of PAL is first induced to increase, followed by an increase in lignin content (Pant and Huang, 2022). CAD is the last enzyme in the lignin synthesis pathway (Moller et al., 2005), and some studies have found that reducing the activity of sorghum CAD enzyme can reduce the content of lignin (Saballos et al., 2009). POD is also one of the key enzymes in the lignin synthesis pathway, which deposits metabolites in plant vessels (Suling, 2001). In this study, the content of lignin and the activities of CAD, PAL, and POD increased gradually after the inoculation of *N. parvum*, and the activities of CAD, PAL, and POD in resistant varieties ‘TF’ blueberry were higher than those in susceptible varieties ‘DF’ blueberry (**Figures 2D**, **3**). This indicated that the blueberry lignin synthesis pathway was involved in resistance to inoculation with *N. parvum* and the lignin biosynthesis pathway of the resistant cultivar ‘TF’ blueberries played a more significant role.

To further understand the role of lignin in blueberry defense against *N. parvum* inoculation, transcriptome sequencing data, we analyzed transcriptome sequencing data in this study. The results of GO enrichment analysis showed that the cell wall macromolecular catabolic process was the most prominent in the biological process of blueberry infected by *N. parvum* within 24 hours, the cell components with the most significant differences observed in the external encapsulating structure, and the chitin activity showing the most variation in the biological function. Of all the components, the most differentially expressed genes were associated with the external encapsulating structure of the cellular components (**Figure 4A**). This indicates that numerous biochemical reactions occur in blueberry cells within 24 hours after inoculation by *N. parvum*, particularly affecting cell wall components. Cellulose and pectin are the main components of plant cell walls, while chitin is the main component of fungal cell walls, and lignin deposition can also lead to changes in plant cell walls. In the process of blueberry resistance to *N. parvum* inoculation, a series of enzymes participate in the response, including peroxidase, which is also the key enzyme of the lignin synthesis pathway. The above results indicated that the inoculation of *N. parvum* could destroy the cell wall of blueberry and affect the activities of various enzymes, thus inhibiting the growth and development of blueberry.

KEGG enrichment analysis revealed 16 KO pathways related to phenylpropanoid biosynthesis, with each pathway corresponding to an enzyme and different enzymes having their corresponding gene regulation (**Figure 4B**). Given the lignin metabolism in the phenylpropanoid biosynthesis pathway, the differentially expressed genes related to phenylpropanoid biosynthesis in tissues of blueberry infected by *N. parvum* are involved in regulation, making the analysis of this pathway crucial for exploring the differentially expressed genes in the lignin biosynthesis pathway of blueberry. Numerous genes were involved in the regulation of lignin synthesis. *PAL*, *COMT*, *CCoAOMT*, *4CL*, *CYP84a*, and *F5H* were down-regulated genes, *CYP73a* and *CCR* were up-regulated genes, and *HCT*, *CYP98a*, *C3’H*, and *REF1* were both up-regulated and down-regulated genes. This indicates that the enzyme activity regulated by the different genes related to the lignin metabolic pathway of blueberries inoculated with *N. parvum* vary, for example, the number of the different genes for *POD* and *CAD* in the two blueberries is more than that of other genes, and both include up-regulated and down-regulated genes. Therefore, the study on the differential gene expression in the lignin synthesis pathway has a further understanding of the gene function and metabolic pathways of blueberry and also provides a reference for the subsequent discovery of disease-resistance genes in blueberry.

### Identification of *CAD* gene family in blueberry and its response to *N. parvum* inoculation

CAD is the final step in lignin biosynthesis and plays a crucial role in plant resistance to biotic stresses such as pathogens through the lignin pathway. A total of 93 *CAD* genes were identified in the blueberry genome level using bioinformatics. In comparison, there are only 9 *CAD* family members in *Arabidopsis thaliana* (Sibout et al., 2005), 12 in rice (Tobias and Chow, 2005), 14 in *Sorghum bicolor* (Saballos et al., 2009), and 43 in wheat (Hao et al., 2022). Blueberry *CAD* family members are more numerous than those in these plants. Phylogenetic analysis revealed that the blueberry *CAD* genes were categorized into four subgroups, and there was no evident regularity in the structure and conserved motifs among the *CAD* genes in the subgroups, while there were significant variations in the length of CDS and the number of conserved motifs. In wheat, 43 *CAD* genes were found to be categorized into 5 subgroups, in which the gene members in subgroup I showed strong conservation in gene structure and conserved motif, and the difference in gene structure of blueberry *CAD* genes may indicate that the conservation among *CAD* genes is low (Hao et al., 2022).

Among the 93 *CAD* genes of blueberry, many are located on the same chromosome, and these genes are located in the same chromosome region, arranged in the form of gene clusters (**Figure 7A**). Some studies have found that lignin synthesis was significantly affected after silencing the expression of a single *CAD* gene, while other studies found that silencing the expression of a single *CAD* gene had no significant effect on lignin synthesis (Mita et al., 2006; An et al., 2024). This phenomenon is primarily due to the expression compensation among the members of the *CAD* gene family. For example, in *Sorghum bicolor*, it was found that when the cinnamyl alcohol dehydrogenase gene *Bmr6* was knocked out, the expression of *SbCAD4* was significantly increased, conversely, in the wild type, higher expression of *Bmr6* corresponded to lower expression of *SbCAD4*. This suggests a complementary effect between the expression of these two genes (Sattler et al., 2009). Some blueberry *CAD* genes are distributed in gene clusters, potentially exhibiting complementary functions, providing a reference for further study of *CAD* gene function.

To validate the blueberry *CAD* gene response to *N. parvum* stress analysis, we conducted QRT-PCR analysis. The results showed that the expression levels of 8 genes in the resistant blueberry ‘TF’ and susceptible blueberry ‘DF’ were significantly increased following *N. parvum* inoculation (**Figure 9**). In soybean, it was observed that the expression of *GmCAD1* and *GmCAD2* was strongly induced in the reproductive period of susceptible and resistant varieties damaged by nematodes, with expression patterns consistent with those of resistant varieties, indicating that *Penicillium purpureum* could induce soybean to produce lignin in response to nematode stress. It is an important molecular mechanism of soybean resistance to nematode stress (Li et al., 2022b). These results suggest that the blueberry *CAD* gene plays an important role in blueberry resistance to *N. parvum* inoculation, and it is a key disease resistance gene, warranting further study.

Our study has preliminarily elucidated the mechanisms of resistance to shoot blight in rabbiteye blueberry cultivars exclusively at the transcriptomic level, with limited validation at the proteomic level. For future research, we plan to employ gene editing technologies to conduct knockout or overexpression analyses on key candidate genes identified in this study, aiming to validate their biological functions at molecular, cellular, and organismal levels. Furthermore, integrative transcriptome-metabolome analyses will be conducted to correlate gene expression profiles with metabolic flux variations, thereby enabling a comprehensive elucidation of metabolic regulatory networks and revealing how transcriptional changes modulate metabolic pathways and metabolite accumulation.

## Conclusion

**Figure 10:**
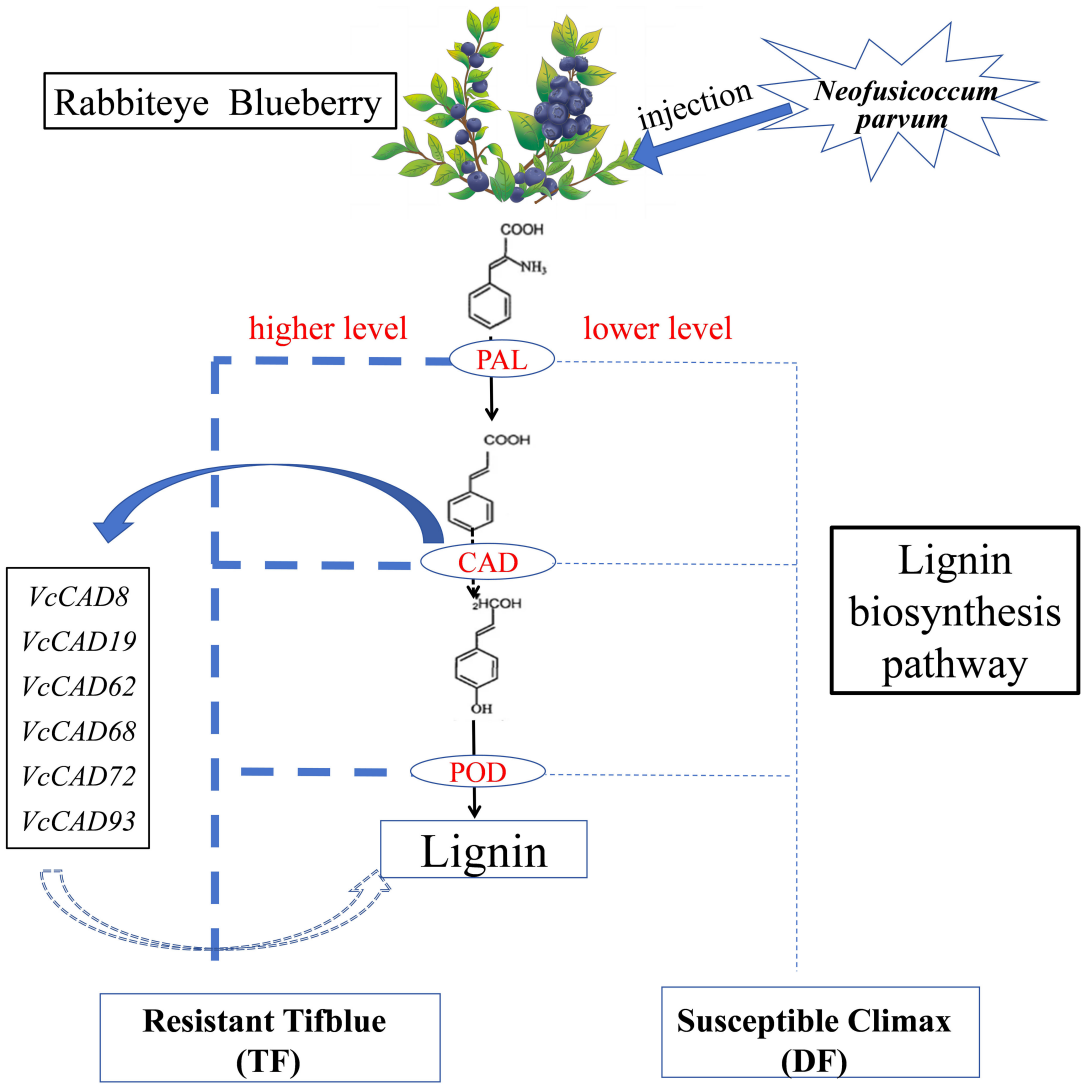
The pathway diagram of *CAD* genes regulating lignin biosynthesis for blueberry inoculated with *N. parvum*.

In this study, we performed comparative physiological, transcriptomic, and gene family analyses on two blueberry varieties (resistant ‘TF’ and susceptible ‘DF’) to *N. parvum* infection. Resistant plants ‘TF’ exhibited significantly higher lignin content and increased activity of key enzymes involved in lignin biosynthesis, including POD, PAL, and CAD. RNA-seq analysis revealed that the phenylpropanoid biosynthesis pathway was central to blueberry resistance, with a marked upregulation of lignin biosynthesis genes in both varieties. Notably, ‘TF’ showed a greater number of DEGs and upregulated genes compared to ‘DF’, with *CAD* and *POD*-related genes being particularly prominent. Further bioinformatics analysis identified and characterized the blueberry *CAD* gene family, providing insights into its evolutionary relationships and genomic organization through phylogenetic and chromosomal localization analyses. Among the *VcCADs* genes, *VcCAD8* and *VcCAD62* were significantly upregulated 24 hours post-infection, suggesting its pivotal role in the defense response. Our findings demonstrated that the *VcCADs* gene family enhances lignin synthesis, thereby improving blueberry resistance to shoot blight. The pathway diagram of *CAD* genes regulating lignin biosynthesis in blueberry is shown in **Figure 10**. This study has preliminarily elucidated the defense mechanisms of blueberries against *Neofusicoccum parvum* and identified key genes in the lignin biosynthesis pathway. Based on these findings, screening for molecular markers associated with disease resistance will facilitate the early and precise identification of disease-resistant plants. Furthermore, integrating these genetic targets with molecular-assisted breeding techniques can accelerate the cultivation cycle of new blight-resistant blueberry varieties, providing technical support for the sustainable development of the blueberry industry.

## Data Availability

The sequence data of this study have been deposited in the National Center for Biotechnology Information with the primary accession code PRJNA1243556.
