# Supplementary material for: Transcriptome and VcCADs gene family analyses reveal mechanisms of blight resistance in rabbiteye blueberry
Source: Front Plant Sci. 2025 Jun 18;16:1601658. doi: 10.3389/fpls.2025.1601658 (PMC12213566; doi:10.3389/fpls.2025.1601658)
Supplement: Supplementary file 1 [file Table1.docx]

**Table S1 Ka/Ks values of duplicate gene pairs**

| Duplicated gene pairs | Ka | Ks | Ka /Ks |
| --- | --- | --- | --- |
| *VcCAD43* and *VcCAD89* | 0.005489503006702356 | 0.037876210638739735 | 0.14493274047556648 |
| *VcCAD43* and *VcCAD15* | 0.005492015387505567 | 0.03352513418843012 | 0.16381784951664474 |
| *VcCAD43* and *VcCAD53* | 0.009635369963690472 | 0.029272007929301423 | 0.3291666901348923 |
| *VcCAD2* and *VcCAD47* | 0.005723580195947717 | 0.03811553710390426 | 0.1501639654281938 |
| *VcCAD2* and *VcCAD75* | 0.0033815574850909573 | 0.03772007958335629 | 0.08964873675884408 |
| *VcCAD28* and *VcCAD41* | 0.0024942862316312503 | 0.003965640435512482 | 0.6289743793448364 |
| *VcCAD28* and *VcCAD14* | 0.002493767883578863 | 0.020054670781424314 | 0.12434848274291899 |
| *VcCAD76* and *VcCAD8* | 0.012402101532169283 | 0.05701523047080613 | 0.2175226063239297 |
| *VcCAD37* and *VcCAD36* | 0.04178370058619428 | 0.14665632616656676 | 0.28490895468592276 |
| *VcCAD89* and *VcCAD15* | 0.002739729074018776 | 0.025037095389904312 | 0.10942679377750483 |
| *VcCAD89* and *VcCAD53* | 0.0068666078179466915 | 0.02505452179962898 | 0.2740666085292586 |
| *VcCAD54* and *VcCAD22* | 0.0056556022545272994 | 0.03326692529488698 | 0.17000676210363655 |
| *VcCAD54* and *VcCAD59* | 0.22236007567623728 | 0.957554949610744 | 0.23221651746109082 |
| *VcCAD15* and *VcCAD53* | 0.009635369963690472 | 0.03784965682251096 | 0.25456954626758643 |
| *VcCAD54* and *VcCAD29* | 0.005653470659003257 | 0.0109391182641695 | 0.5168122807046432 |
| *VcCAD51* and *VcCAD72* | 0.02233326255355231 | 0.01131669905225105 | 1.973478525004154 |
| *VcCAD31* and *VcCAD8* | 0.01686165940007319 | 0.06757637602288988 | 0.2495200304076932 |
| *VcCAD22* and *VcCAD59* | 0.21708110069062947 | 0.9374487274882787 | 0.2315658385629882 |
| *VcCAD22* and *VcCAD29* | 0.0022556407979664964 | 0.02956047640565747 | 0.07630596905856353 |
| *VcCAD11* and *VcCAD56* | 0.0027485142991109423 | 0.009063554411303914 | 0.3032490537799432 |
| *VcCAD11* and *VcCAD85* | 0.020734972919162705 | 0.03294897525903407 | 0.6293055476278436 |
| *VcCAD64* and *VcCAD1* | 0.004844586889162179 | 0.04033229758560305 | 0.12011680908779904 |
| *VcCAD64* and *VcCAD74* | 0.004848501734107419 | 0.048534450519275234 | 0.09989814826855531 |
| *VcCAD59* and *VcCAD29* | 0.2206202452943778 | 0.9484900084277266 | 0.2326015491297489 |
| *VcCAD64* and *VcCAD82* | 0.15711314996886772 | 1.4592701334546225 | 0.10766556949735126 |
| *VcCAD84* and *VcCAD65* | 0.06346324646062895 | 0.11162895558572145 | 0.5685195756570044 |
| *VcCAD84* and *VcCAD79* | 0.0048919863615625656 | 0.019974549424199208 | 0.24491097434397763 |
| *VcCAD46* and *VcCAD33* | 0.005472479588152162 | 0.030813062527406166 | 0.17760258602288417 |
| *VcCAD46* and *VcCAD42* | 0.0022134111795016525 | 0.03515124277063031 | 0.06296821975668553 |
| *VcCAD1* and *VcCAD74* | 0.003634902106897244 | 0.05265319400493634 | 0.06903478840346255 |
| *VcCAD1* and *VcCAD82* | 0.16142431176438132 | 1.431244580776843 | 0.11278597238549146 |
| *VcCAD74* and *VcCAD82* | 0.15657827721721249 | 1.455396872393681 | 0.10758459097118252 |
| *VcCAD41* and *VcCAD14* | 0.0049979360298735235 | 0.02409845863709488 | 0.2073965022053392 |
| *VcCAD20* and *VcCAD3* | 0.015125067750639935 | 0.03500004532329192 | 0.4321442332697342 |
| *VcCAD20* and *VcCAD71* | 0.017515143913239196 | 0.03895745862063034 | 0.4495966763079321 |
| *VcCAD20* and *VcCAD27* | 0.016914663496854814 | 0.06072679690025032 | 0.27853706041237114 |
| *VcCAD3* and *VcCAD71* | 0.00464757490313362 | 0.04131206931358559 | 0.112499203752189 |
| *VcCAD3* and *VcCAD27* | 0.006399108045924682 | 0.04321796614554699 | 0.1480659229630134 |
| *VcCAD17* and *VcCAD36* | 0.0022975319518036877 | 0.034335302590340455 | 0.0669145683443038 |
| *VcCAD70* and *VcCAD6* | 0.0012192646456874433 | 0.019545080002398716 | 0.06238217728133147 |
| *VcCAD70* and *VcCAD45* | 0.0036659950791660895 | 0.007742004230564043 | 0.4735201596368805 |
| *VcCAD69* and *VcCAD78* | 0.001352875225909325 | 0.012866648653486773 | 0.1051458901493129 |
| *VcCAD7* and *VcCAD12* | 0.004822198649670304 | 0.05304725680133928 | 0.09090382689776634 |
| *VcCAD6* and *VcCAD45* | 0.004889992872960896 | 0.019532353902014986 | 0.2503534851709009 |
| *VcCAD6* and *VcCAD78* | 0.03737366430434588 | 0.30024278145580885 | 0.12447814439744229 |
| *VcCAD45* and *VcCAD21* | 0.004890989414132581 | 0.01951964436409954 | 0.25056754738462705 |
| *VcCAD5* and *VcCAD78* | 0.0027075841680747554 | 0.012875852716774085 | 0.21028387227103304 |
| *VcCAD71* and *VcCAD27* | 0.00873457077193857 | 0.07093405504090343 | 0.12313649299905192 |
| *VcCAD33* and *VcCAD42* | 0.0022134111795016525 | 0.003825319099244096 | 0.5786213181376253 |
| *VcCAD65* and *VcCAD79* | 0.06489591334331607 | 0.11121980416860253 | 0.5834924259076897 |
